# Implementing antiretroviral resistance testing in a primary health care HIV treatment programme in rural KwaZulu-Natal, South Africa: early experiences, achievements and challenges

**DOI:** 10.1186/1472-6963-14-116

**Published:** 2014-03-07

**Authors:** Richard J Lessells, Katharine E Stott, Justen Manasa, Kevindra K Naidu, Andrew Skingsley, Theresa Rossouw, Tulio de Oliveira

**Affiliations:** 1 Africa Centre for Health and Population Studies, University of KwaZulu-Natal, PO Box 198, Mtubatuba, KwaZulu-Natal 3935, South Africa; 2 Department of Clinical Research, London School of Hygiene and Tropical Medicine, London, UK; 3 Department of Infectious Diseases, Imperial College, London, UK; 4 Department of Family Medicine, Faculty of Health Sciences, University of Pretoria, 7th Floor, HW Snyman North building, Prinshof Campus, Pretoria, South Africa; 5 Research Department of Infection, University College London, London, UK

**Keywords:** HIV-1, Drug resistance, Anti-retroviral agents, Primary health care, Treatment failure, Process assessment (health care), Capacity building

## Abstract

**Background:**

Antiretroviral drug resistance is becoming increasingly common with the expansion of human immunodeficiency virus (HIV) treatment programmes in high prevalence settings. Genotypic resistance testing could have benefit in guiding individual-level treatment decisions but successful models for delivering resistance testing in low- and middle-income countries have not been reported.

**Methods:**

An HIV Treatment Failure Clinic model was implemented within a large primary health care HIV treatment programme in northern KwaZulu-Natal, South Africa. Genotypic resistance testing was offered to adults (≥16 years) with virological failure on first-line antiretroviral therapy (one viral load >1000 copies/ml after at least 12 months on a standard first-line regimen). A genotypic resistance test report was generated with treatment recommendations from a specialist HIV clinician and sent to medical officers at the clinics who were responsible for patient management. A quantitative process evaluation was conducted to determine how the model was implemented and to provide feedback regarding barriers and challenges to delivery.

**Results:**

A total of 508 specimens were submitted for genotyping between 8 April 2011 and 31 January 2013; in 438 cases (86.2%) a complete genotype report with recommendations from the specialist clinician was sent to the medical officer. The median turnaround time from specimen collection to receipt of final report was 18 days (interquartile range (IQR) 13–29). In 114 (26.0%) cases the recommended treatment differed from what would be given in the absence of drug resistance testing. In the majority of cases (n = 315, 71.9%), the subsequent treatment prescribed was in line with the recommendations of the report.

**Conclusions:**

Genotypic resistance testing was successfully implemented in this large primary health care HIV programme and the system functioned well enough for the results to influence clinical management decisions in real time. Further research will explore the impact and cost-effectiveness of different implementation models in different settings.

## Background

The rapid expansion of public health human immunodeficiency virus (HIV) programmes in the past decade has led to over eight million people accessing antiretroviral therapy (ART) in low- and middle-income countries [1]. Many challenges remain as we transition from an emergency response to long-term, sustainable strategies [2,3], one of which is the prevention and management of antiretroviral drug resistance [4].

The World Health Organization (WHO) global strategy on drug resistance relies on surveillance of transmitted and acquired resistance, with survey results informing treatment policies [5,6]. In time, the detection of drug resistance for individual case management might become more important as the public health approach to ART delivery becomes less effective and as the case mix becomes more complex. In South Africa, the national antiretroviral treatment guidelines now incorporate recommendations for genotypic resistance testing in certain situations (e.g. failure of second-line ART in adults and children) [7]. The Southern African HIV Clinicians Society guidelines go further in recommending genotypic resistance testing at time of first-line ART failure in adults [8].

The Southern African Treatment and Resistance Network (SATuRN) has developed models for implementation of antiretroviral drug resistance testing within HIV programmes, with the aim of integrating clinical care, training, surveillance and research [9,10]. Different implementation models have so far been introduced within the national programme in Botswana and in the Free State province of South Africa. Here we describe an HIV Treatment Failure Clinic (HIV-TFC) model developed for a primary health care programme in rural KwaZulu-Natal. We have reported clinical outcomes for adults on ART in this programme that are broadly similar to other public sector programmes in South Africa [11-13]. However, the programme’s systems for detection and management of ART failure have been relatively ineffective, to the extent that, of the 20 000 adults enrolled on ART by the end of 2010, fewer than 100 (0.5%) had been switched to second-line ART regimens despite virological failure rates comparable to other programmes in the region [14]. The implementation of antiretroviral drug resistance testing as part of a research study provided an opportunity to address the backlog of treatment failure cases and provide focused training to staff on the detection and management of treatment failure, whilst simultaneously facilitating drug resistance surveillance and research. We have reported the detailed results from the antiretroviral drug resistance testing for adults separately [15]. Here we focus on a quantitative process evaluation of the system and a discussion of the main achievements and challenges of implementation.

## Methods

### Setting

The Hlabisa HIV Treatment and Care Programme has since August 2004 provided comprehensive HIV services at 17 primary health care (PHC) clinics and one district hospital in the predominantly rural Hlabisa sub-district (1430 km^2^) in northern KwaZulu-Natal [11,12]. The programme is co-ordinated by the local Department of Health with support from the Africa Centre for Health and Population Studies (Africa Centre, http://www.africacentre.ac.za). HIV treatment and care is delivered largely by nurses and counsellors, with medical officers visiting clinics on a weekly or fortnightly basis. The programme adheres to national antiretroviral treatment guidelines and routine laboratory monitoring (CD4+ cell count and HIV-1 viral load) is performed at the National Health Laboratory Service (NHLS) laboratory situated at the district hospital. The scale-up of antiretroviral therapy has been rapid such that, by the end of 2012, more than 50,000 individuals had been enrolled in HIV care and 25,000 individuals had initiated ART.

### Participants

HIV-infected adults (≥16 years) with virological failure on first-line (non-nucleoside reverse transcriptase inhibitor (NNRTI)-based) ART were eligible for the intervention. Virological failure was defined for this study as one viral load >1000 copies/ml after at least 12 months of first-line ART. The intervention was initially piloted from December 2010 and was fully operational by April 2011. Individuals were recruited at all 17 PHC clinics. We implemented the HIV-TFC model in two different strategies at the clinic level, with the aim of allowing future comparison of patient outcomes and costs. The first involved patients being enrolled for resistance testing by the medical officer during routine clinic visits, with referral to members of the multidisciplinary team such as the social worker, the psychologist or the dietician as appropriate, on a case-by-case basis. The second strategy adopted a treatment failure support camp approach, whereby eligible individuals were identified from the programme database; individuals were then invited in groups of 10 to 15 to attend their normal clinic on a specific day and each participant was reviewed by a social worker and a community adherence educator in addition to the medical officer, in order to specifically address adherence issues, with referral to other multidisciplinary team members on the same day if required.

### Intervention

**Figure 1:**
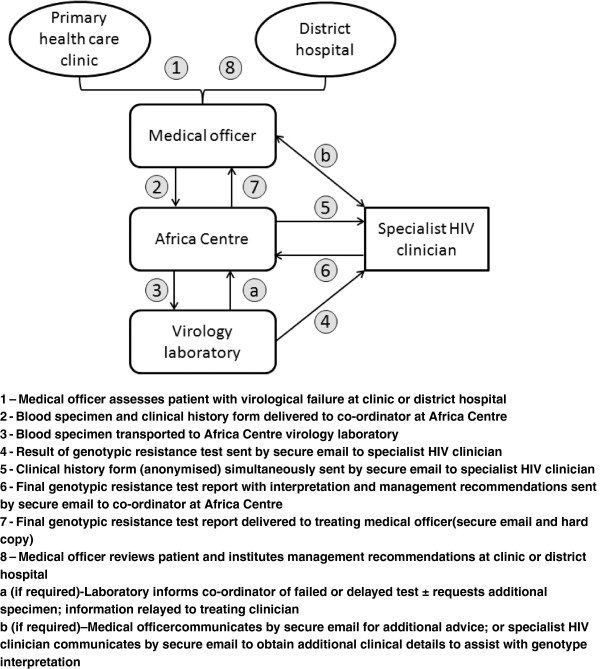
The HIV Treatment Failure Clinic (HIV-TFC) model.

The intervention model for incorporating resistance testing into routine clinic management is illustrated in Figure 1. At the initial clinic visit, the medical officer obtains written informed consent for the study, performs a clinical assessment and completes a standardised clinical history form. A blood sample is collected by the medical officer at the clinic for HIV drug resistance genotyping and is transported to the Africa Centre (mean distance 30km, range 2–68) and then onwards the same day to the laboratory in Durban (~250 km). The clinical history form is delivered to the Africa Centre by the medical officer and these data are anonymised and captured into a relational database (SATuRN RegaDB) [10,16,17]. Genotypic resistance testing is performed using the in-house SATuRN/Life Technologies method [18]. In order to keep the costs low, no viral load was performed before drug resistance genotyping. A genotype report is generated in the laboratory and an initial ‘plain-language’ summary is added by laboratory staff. This initial report is then sent via email with the clinical history form to a specialist HIV clinician, who provides a more specific interpretation with recommendations for on-going medical management, including antiretroviral regimen change where appropriate. As far as possible, recommendations for second-line regimens are made in accordance with national guidelines, with deviations suggested only when thought to be absolutely necessary. This final report is emailed to the medical officer and to the data curation team for entry into the SATuRN RegaDB. The patient then attends their normal clinic for review and treatment is changed as appropriate. Requests for standardised second-line regimens are approved by the pharmacists at the district hospital whereas requests for non-standard regimens are submitted to and approved by a provincial task team. The model aims for a turnaround time, from collection of clinical information and blood sample to receipt of final report by the medical officer, of fewer than 30 days.

### Training, education and capacity building

All medical officers involved in the delivery of HIV care at the PHC clinics received basic training in antiretroviral treatment failure, drug resistance and the implementation model prior to commencement of the project. The education and training of nurses and counsellors at the clinics was then performed in-situ by the doctors during routine clinic visits and continued throughout the project. Community education was facilitated by a two-day workshop with members of the Africa Centre Community Engagement Office and Community Advisory Board. Selected clinic staff attended a drug resistance training workshop at the Africa Centre in May 2012 to consolidate training – this used a case-based learning approach. Key programmatic personnel also attended the SATuRN workshops in October 2010, October 2011 and November 2012. In addition, a HIV & TB drug resistance case book was launched at a workshop in March 2013 and was distributed to all medical officers and selected clinic nurses [19].

### Process evaluation

A quantitative process evaluation was conducted alongside the implementation of the programme. The aims of this process evaluation were to determine the extent to which the programme was implemented according to the protocol and to aid explanation of programme outcomes through analysis of operational successes and failures. The key components of the process evaluation and the data sources are displayed in Table 1. Contemporaneous records of the processes were maintained by the co-ordinating team at the Africa Centre and by the laboratory. All email communication (including reports sent to and received from the specialist clinicians) was stored in secure email archives. Medical officers updated the co-ordinating team with details of antiretroviral switches. Where no follow-up information was available from the medical officer, clinic files were reviewed to ascertain vital status and relevant treatment information. All follow-up data capture was completed by 30 April 2013, thus allowing at least three months post-genotype for all participants.

**Table 1:** Indicators for process evaluation

**Indicator**	**Data source**	**Comments**
Uptake of testing (by medical officer and by clinic)	Study and laboratory records	Numbers enrolled by medical officer and by clinic
Proportion with valid genotype result	Laboratory records	Excludes instances of failed amplification or sequencing
Proportion with initial laboratory report	Laboratory records	Genotype report generated within laboratory
Proportion sent to specialist clinician	Laboratory email records	Genotype report sent by email to specialist clinician
Proportion with final laboratory report	Laboratory email records	Final report (with specialist clinician comments) issued by laboratory
Turnaround time (laboratory, specialist clinician, overall)	Study, laboratory and specialist clinician records	Laboratory – time from receipt of specimen to initial genotype result; specialist clinician – time from receipt of initial genotype result to generation of final laboratory report
Proportion switched to second-line regimen as recommended	Study and medical officer records; clinic file review	Concordance with specialist clinician recommendations

### Ethical issues

The study was approved by the Biomedical Research Ethics Committee of the University of KwaZulu-Natal (BF052/10) and the Health Research Committee of the KwaZulu-Natal Department of Health (176/10). The study was also approved by the Community Advisory Board of the Africa Centre and permission for entry into healthcare facilities was obtained from Hlabisa Hospital. Written informed consent was obtained from all participants. There was no financial incentive or reimbursement for participation as procedures were part of routine clinical care.

## Results

### Uptake of intervention

All patients approached to participate in the study accepted the offer of a resistance test. In total, 508 specimens were submitted for genotyping between 8 April 2011 and 31 January 2013. Ten medical officers were trained and actively involved in managing patients within the treatment failure clinic system. The number of resistance tests requested per medical officer varied substantially, due in part to varied lengths of service within the programme and in part to differences in doctors’ roles in relation to the intervention: those medical officers who requested resistance tests opportunistically in primary health care clinics enrolled between 17 and 32 participants each, whereas two medical officers who were more directly involved in the treatment failure support camps enrolled 168 and 99 participants respectively. The medical officers themselves ranged from those at an early stage of their careers (post-internship) to HIV specialists with several years of experience in the local setting. Adult patients from all 17 PHC clinics in the Hlabisa sub-district were enrolled, with between one and 193 specimens originating from the different clinics.

### Production of resistance reports

**Figure 2:**
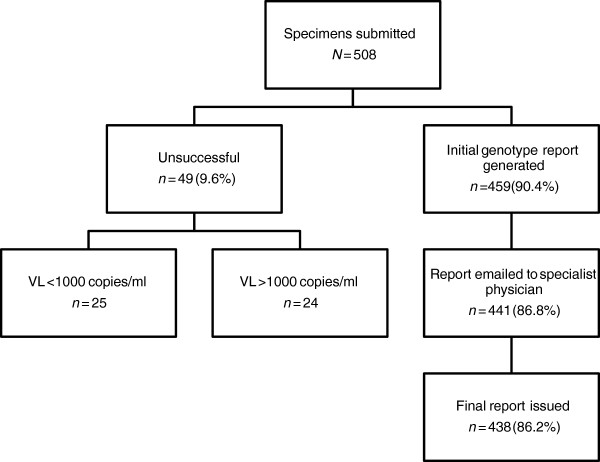
Flow diagram of processes involved in production of final resistance report.

Of the 508 genotypes requested, 459 (90.4%) had successful amplification and sequencing in the laboratory (Figure 2). Of the 49 specimens that did not amplify, 25 subsequently had a viral load measurement on the same specimen of <1000 copies/ml. For all specimens that were successfully amplified, an initial laboratory report was produced. 441 of the 459 reports were emailed to one of the specialist HIV clinicians, who returned a final report, including any recommendations for ART regimen change, for 438 of these cases.

Overall, the median turnaround time from specimen collection to receipt of final report was 18 days (interquartile range (IQR) 13–29). The most time-consuming step was the genotypic resistance testing itself, which took a median of 12 days (IQR 7–20). Where there were substantial delays in this step (> 50 days from sample date to genotype production, *n* = 27), this was usually due to the specimen requiring more than one attempt to amplify. The median time taken by the specialist HIV clinician to append the laboratory report with specific interpretation and management recommendations and to send the final report back to the medical officer was two days (IQR 1–6).

Analysis of process indicators pertaining to the clinic versus support camp models suggested a less efficient process of genotypic resistance test report generation for the support camps, although in both contexts the median overall turnaround time for report production was under the target of 30 days (Table 2). The proportion of samples that were successfully amplified in each context was similar.

**Table 2:** Process indicators: production of resistance report

	Genotype generation*	**Laboratory report†**	**Specialist recommendations‡**	**Total report turnaround time§**
**All samples**				
Median	12	1	2	18
IQR	7-20	0-3	1-6	13-29
Range	0-151	0-61	0-24	2-154
**Clinic**				
Median	12	1	2	16
IQR	8-19	0-3	1-5	13-28
Range	0-151	0-61	0-24	4-154
**Support camp**				
Median	15	1	4	25
IQR	7-23	0-2	2-7	13-29
Range	0-144	0-47	0-17	2-153

IQR, interquartile range.

*Time from specimen collection to generation of initial genotype report.

^†^Time from generation of initial genotype report to sending report to specialist clinician.

^‡^Time from sending of laboratory report to specialist clinician to receipt of final report by medical officer.

^§^Time from initial specimen collection to receipt of final report by medical officer.

### Treatment recommendations and patient follow up

Of the 438 genotypes for which a complete resistance report was produced, 50 (11.4%) had no major drug resistance-associated mutations (DRAMs) and the treatment recommendation in 49 of those cases was to continue first-line ART. A standard second-line regimen included in the national antiretroviral guidelines was recommended in the presence of DRAMs for 365 (83.3% of total): for 324 (74.0% of total) this was the second-line regimen that would have been prescribed in the absence of resistance testing; whereas in 41 cases (9.4% of total), an alternative standard second-line regimen was recommended based on the pattern of mutations and drug history. For example, zidovudine (AZT), lamivudine (3TC) and lopinavir/ritonavir (LPV/r) was recommended for patients failing on a stavudine (d4T)-based first line regimen with the K65R mutation, or tenofovir (TDF), 3TC and LPV/r was recommended for a patient failing a TDF-based first line regimen with only NNRTI resistance mutations). For 21 patients (4.8%), a non-standard second-line regimen, consisting of second-line drugs contained within the guidelines, was recommended: one example was three nucleoside reverse-transcriptase inhibitors with LPV/r (for reasons other than hepatitis B infection). Finally, for three patients, a non-standard regimen containing at least one third-line antiretroviral agent (e.g. raltegravir (RAL) or etravirine (ETR)) was recommended.

Follow-up information was recorded for 412 participants (94.1%). Table 3 demonstrates the concordance between report recommendations and actual prescribed ART regimens for participants. 312 patients (75.7%) were on second-line ART regimens, 276 (88.5%) of these in direct accordance with the specialist HIV clinician’s recommendations. In cases where the second-line regimen prescribed contravened the specialist recommendations (*n* = 36), this was most commonly due to contraindications to particular antiretroviral drugs (e.g. renal impairment or anaemia) that were not known or not documented at the time of the initial clinical assessment. As described, three resistance reports included recommendation of a regimen including one or more third-line antiretroviral, due to the presence of multinucleoside resistance mutations (e.g. Q151M complex). In all of these cases, the recommended regimen was RAL, ETR, 3TC and LPV/r. Two of these patients received RAL, 3TC and LPV/r (without ETR) but in the third case the treatment switch was delayed due to severe illness and the patient died from disseminated tuberculosis a week after the final resistance report was produced. In 26 cases overall (5.9%), there was no documentation of a follow-up visit or a treatment switch.

**Table 3:** Specialist recommendations and follow-up outcomes

**Specialist recommendation**	***n***	**Regimen prescribed matched regimen recommended by specialist**	**Regimen prescribed did not match regimen recommended by specialist**	**Continued first-line regimen despite recommendation for switch**	**Data missing**
Standard second-line ART*	324	247 (76.2)	17 (5.2)	39 (12.0)	21 (6.5)
**Other standard second-line ART†**	41	22 (53.7)	9 (22.0)	8 (19.5)	2 (4.9)
**Non-standard second line ART‡**	21	7 (33.3)	10 (47.6)	1 (4.8)	3 (14.3)
**Non-standard salvage regimen§**	3	0	2 (66.7)	1 (33.3)	0
**Continue first-line ART**	49	39 (79.6)	10 (20.4)	NA	0
**Total**	**438 (100)**	**315 (71.9)**	**48 (11.0)**	**49 (11.2)**	**26 (5.9)**

*Second-line regimen recommended by national guidelines in the absence of genotypic resistance testing (based on treatment history).

^†^Second-line regimen included in national guidelines but not the regimen that would have been prescribed based on treatment history.

^‡^Alternative second-line regimen incorporating only drugs in national guidelines (e.g. 3 nucleoside reverse-transcriptase inhibitors and protease inhibitor).

^§^Alternative regimen incorporating novel antiretroviral agents (e.g. raltegravir, etravirine).

## Discussion

This paper describes our HIV-TFC model for management of ART failure incorporating the use of genotypic resistance testing within a PHC programme in rural KwaZulu-Natal. With this model we have managed 508 adults with virological failure on first-line ART between April 2011 and January 2013. The majority (76%) of those patients are now on appropriate second-line ART regimens.

Several others (9%) have avoided inappropriate switch to second-line therapy after genotypic evidence of susceptibility to first-line ART and have instead received intensified adherence support (the effect of which can be seen in Case example 1). Medical officers were able to manage patients with complex antiretroviral resistance patterns in spite of limited experience in this specialty, due to the clarity of genotypic interpretation and input of specialist HIV clinicians with resistance reports. This provides some evidence of the feasibility of genotypic resistance testing for patients with ART failure in a rural and relatively resource-limited South African context.

### Case example 1

**Figure 3:**
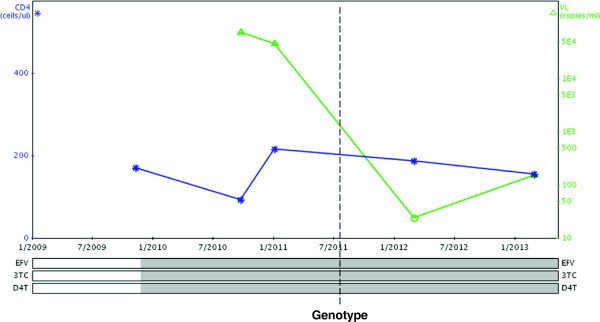
Graphical display of antiretroviral history and viral load/CD4+ cell count measurements (Case example 1).

A 54-year-old male was seen in July 2011 with virological failure on a first-line regimen consisting of stavudine (d4T), lamivudine (3TC) and efavirenz (EFV). He had commenced treatment in November 2009 with WHO clinical stage 3 (concurrent pulmonary TB) and baseline CD4+ cell count of 172 cells/μL. There was no evidence of virological suppression after 10 months of treatment and, after intensive adherence counselling, viral load remained elevated at 14 months (Figure 3). Objective assessment suggested good adherence and he had been on time for each monthly clinic visit and pharmacy refill. His wife was also on ART and had full virological suppression and he reported that they supported each other with treatment. He did, however, disclose use of over-the-counter ‘immune boosters’. Genotypic resistance test demonstrated no specific mutations (i.e. the predominant viral strain was wild type). He was maintained on the same regimen (d4T/3TC/EFV) and received further intensive adherence support and counselling, with particular focus on the benefits of ART and the risks of using non-prescribed medication. Six months later, viral load was suppressed (<40 copies/ml) and this was maintained <400 copies/ml after a further 12 months, suggesting sustained improvement in adherence to ART.

Overall, we found that most participants harboured HIV strains with at least one drug resistance-associated mutation, although in keeping with other studies around 10% had no evidence of drug resistance [20-23]. In around one in four patients overall (114/438, 26.0%), the results of genotypic resistance testing significantly influenced clinical management, either by allowing continuation of the first-line regimen or by informing the selection of an appropriate second-line regimen that was different to the regimen that would have been recommended in the absence of genotypic resistance testing (see Case example 2). Economic models have suggested that, in the South African context, resistance testing would have important clinical impacts and be cost-effective in adults at first-line ART failure [24]. The proportion with wild type genotype reported here (11%) is close to the threshold at which resistance testing would be very cost-effective according to one model [24]. However, determining the true clinical impact and cost-effectiveness of resistance testing in low-income and middle-income settings will require well-designed clinical trials.

### Case example 2

**Figure 4:**
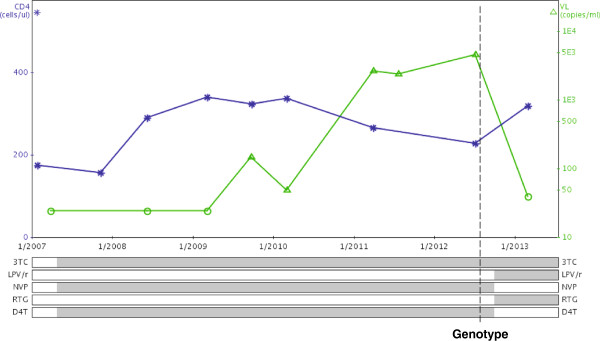
Graphical display of antiretroviral history and viral load/CD4+ cell count measurements (Case example 2).

A 33-year-old female was seen in July 2012 with virological failure on a first-line regimen of stavudine (d4T), lamivudine (3TC) and nevirapine (NVP). She had commenced treatment in April 2007 with baseline CD4+ cell count of 176 cells/μL. There was an excellent initial virological response to ART accompanied by modest immune recovery. Low-level viral rebound was then followed by high-level viraemia which persisted for over 12 months without switch to second-line therapy (Figure 4). She admitted that her adherence had lapsed around the time of the deaths of two close family members. Genotypic resistance test demonstrated extensive drug resistance – in particular the Q151M complex (Q151M + V75I + F116Y), K65R and M184V which together confer high-level resistance to all nucleoside/nucleotide reverse-transcriptase inhibitors. As a standard second-line regimen would only contain a single active drug (lopinavir/ritonavir (LPV/r), a request was made for special access to raltegravir (RTG), to form a regimen of RTG/3TC/LPV/r. This request was approved by the provincial Department of Health and she was commenced on this regimen in October 2012. After four months of this regimen, adherence was excellent and viral load was <40 copies/ml.

The process evaluation revealed a number of systematic challenges. We found that the generation of genotypes was labour intensive and time consuming [18]. This contributed substantially to the overall turnaround time and required that patients be scheduled for follow up one month after initial clinic visit. Whilst this month allowed time for intensified adherence counselling, it might also have provided opportunities for clinical progression and disengagement from care. In the absence of our model, patients with two consecutive viral loads > 1000 copies/ml could have been switched to second-line therapy on their first visit as per national guidelines, assuming that adherence issues had already been addressed. It is possible that the system might have adversely affected the clinical management of patients who had delayed switching, although we have no direct evidence to support this. However, we have shown that in over one quarter of patients a switch to standard second line therapy might be inappropriate or lead to suboptimal outcomes. In addition, medical officers attended the majority of clinics on a regular basis so that there were many opportunities for re-referral upon patients’ subsequent visits. Whilst access to non-standard second-line regimens was possible through application to a provincial specialist panel, this process also often gave rise to substantial delays. We also demonstrated how the second-line regimen sometimes differed from that recommended by the specialist clinician and this was most commonly due to new information becoming available between genotyping and time of treatment switch (e.g. discovery of kidney disease and contra-indication to tenofovir). This demonstrates the need for clear communication channels so that advice can be modified in light of additional information.

Aside from these issues, the model has produced major successes in a challenging environment. A genotypic resistance report was generated from more than 90% of all specimens sent. The specialist HIV clinicians amended the reports promptly and comprehensively so that patients in rural clinics, usually served by one medical officer visiting on a weekly or fortnightly basis, received input from experts in HIV clinical care, which also often went beyond just the ART recommendation. This model also allowed patients to be treated in the PHC setting and avoid travelling to a reference centre in Durban (approximately 250 km) in order to receive drug resistance genotypic testing and specialist support. This application of telemedicine afforded convenience to both the specialist and the medical officer and enabled advanced care of hundreds of patients in a relatively efficient manner. Whilst this has so far involved paper-based reports, as the PHC clinics lack computers and some have erratic electricity supply, there would be potential in the future to develop the model fully around electronic health (eHealth) and mobile health (mHealth) technologies [25].

Of particular relevance to the replication of this model in other contexts are the findings that the process operated more rapidly in the clinic than in the support camp setting, and that rates of follow-up were not significantly different between the two. This could imply that the more natural of the two applications of the model, which is more easily integrated into existing HIV clinics, is the more efficient one, although whether or not this would be the case outside of a research study under routine clinical and laboratory systems remains to be seen. It could alternatively be due largely to capacity limitations and the large number of samples produced in bulk with the support camp system may have overloaded the lab and the specialist clinician. However, the support camp system has additional benefits, such as the opportunity for group counselling and education sessions and multidisciplinary input and whether or not these translate to better clinical outcomes and cost-effectiveness is something that is being explored in additional analyses.

The resistance test reports served not only as tools for clinical management but also for education and training. Medical officers and nurses involved in the system were able to learn about drug resistance patterns and their management in an applied environment. More generally, awareness of the problem of antiretroviral resistance and of issues relating to prevention of drug resistance was raised amongst medical officers, nurses, counsellors and patients. However, the ongoing programme of work relating to antiretroviral resistance in this area means that medical expertise, resources and opportunities for training exist that might not elsewhere in decentralised programmes. There is therefore a need now to explore the operational feasibility of resistance testing in different programmatic settings and to determine the key requirements for successful implementation.

During the implementation of the intervention we encountered a number of problems which we attempted to address as the model evolved. We now have a system within which the processes are almost always successfully operationalised, enabling individualised management of complex resistance cases, employing expert consultation via telemedicine and utilising multidisciplinary referral as appropriate. The system raises awareness amongst frontline health care workers of the increasing threat of drug resistance and our aim is to strengthen capacity within the public health system to manage and monitor the delivery of ART. This model also provides valuable real-time data for surveillance of drug resistance which, through the use of SATuRN RegaDB, provides a highly valuable tool for patient management, clinical training, research and surveillance to inform policy making [10].

## Conclusions

In this paper we have demonstrated the feasibility of a HIV-TFC model in a large PHC ART programme. Given the relatively high proportion of patients for whom standard second-line ART regimens might be suboptimal, it seems that the public health approach to the management of treatment failure and switch to second-line ART might have some limitations in this setting. Resistance testing at treatment failure is routine in the US and Europe, and has recently been recommended in certain situations for South Africa [7,8]. Here we have reported only on a single programme and the fact that the system was implemented as part of a research project with additional human and other resources does somewhat limit the generalisability of our findings. Implementation within the public health systems of southern Africa would be a massive challenge to clinical and laboratory systems and it is important that further research is performed to explore the feasibility, impact and cost-effectiveness of different implementation models within public health systems.

## Abbreviations

3TC: Lamivudine; ART: Antiretroviral therapy; AZT: Zidovudine; CAB: Community advisory board; d4T: Stavudine; ETR: Etravirine; HIV: Human immunodeficiency virus; LPV/r: Lopinavir/ritonavir; NHLS: National Health Laboratory Service; NNRTI: Non- nucleoside reverse transcriptase inhibitor; NRTI: Nucleoside reverse transcriptase inhibitor; PHC: Primary health care; PI: Protease inhibitor; RTG: Raltegravir; SATuRN: Southern African Treatment and Resistance Network; TDF: Tenofovir; TFC: Treatment failure clinic; WHO: World Health Organization.

## Competing interests

The authors declare that they have no competing interests.

## Authors’ contributions

RJL, JM, KKN, AS and TdO conceived and designed the original study. KES contributed to implementation and assisted with the process evaluation. TR provided specialist advice to guide implementation. RJL and TR fulfilled the role of specialist HIV clinician. RJL wrote the first draft of the manuscript. All authors contributed to revision of the manuscript and approved the final version.

## Authors’ information

TdO is the Director and RJL is an Executive Committee member of the Southern African Treatment and Resistance Network (SATuRN).

## Pre-publication history

The pre-publication history for this paper can be accessed here:

http://www.biomedcentral.com/1472-6963/14/116/prepub
